# A Case of Cold Urticaria Diagnosed in the Emergency Department

**DOI:** 10.7759/cureus.84404

**Published:** 2025-05-19

**Authors:** Philip Carhart, James Espinosa, Frank Wheeler, Alan Lucerna

**Affiliations:** 1 Emergency Medicine, Jefferson Health, Stratford, USA

**Keywords:** cold stimulation test, cold urticaria, emergency department diagnosis of cold urticaria, emergency department presentation of cold urticaria, emergency department use of cold stimulation test

## Abstract

Cold urticaria can be primary (idiopathic) or secondary due to underlying hematologic or infectious diseases. Here, we present the case of a 19-year-old female patient with no past medical history who was diagnosed with cold urticaria in the emergency department (ED) setting using a cold stimulation test. Most cases are idiopathic. The reaction can be triggered in individual cases by exposure to cold objects or to generalized cold ambient temperatures, as was the case in the patient presented here. The physical response is most commonly pruritic wheals (urticaria). However, more severe symptoms may occur, up to angioedema with hoarseness and wheezing. This patient had mild symptoms, affecting the skin only. The treatment is essentially symptomatic for mild cases, involving non-sedating histamines. Patient education concerning avoiding cold aquatic activities is important. Anaphylaxis is treated as indicated. ED management of mild cases may include steroid administration. Several sources refer to the consideration of the use of omalizumab in chronic cases.

## Introduction

Cold urticaria is a form of physically induced urticaria, and it presents as cold-induced wheals in response to direct or indirect exposure to cold [1]. The physical response is most commonly pruritic wheals (urticaria). However, more severe symptoms may occur, including more generalized urticaria, angioedema, wheezing, hypotension, and tachycardia. Shock has been reported in susceptible patients who were exposed to cold water while swimming [2]. Cold environmental exposure during surgery has been reported as a trigger. Anaphylaxis from exposure to cold ambient weather has been reported [2].

The incidence of cold urticaria has been estimated to be 0.05% of the population. Most pediatric studies of cold urticaria have shown an equal distribution between males and females, whereas most adult studies have shown a female predominance [1,3].

There are various ways of subdividing forms of cold urticaria. A common approach divides cold urticaria into primary (idiopathic) and secondary (acquired) forms. Some infectious diseases, such as infectious mononucleosis and streptococcal pharyngitis, have been implicated. Malignancies, especially leukemia, have caused cold urticaria [4]. Some vasculitides, such as systemic lupus erythematosus and rheumatoid arthritis, have been implicated. Griseofulvin has been associated [4].

Another way of subtyping cold urticaria is by typical versus atypical symptoms [3]. Typical symptoms show a localized response of the development of a pruritic wheal to localized cold stimulation, with no systemic symptoms. Atypical symptoms show a localized response to cold stimulation associated with systemic symptoms such as wheezing, generalized urticaria, angioedema, and even anaphylaxis. Cold urticaria is said to be chronic when symptoms last longer than six weeks [4].

## Case presentation

A 19-year-old female patient with no past medical or surgical history presented to the emergency department (ED) complaining of an itchy rash on her face, lower extremities, and ears for a three-hour duration. She also reported scratchiness in her throat over the same time duration. She denied exposure to known allergens, new soaps, detergents, perfumes, foods, or medications. She denied difficulty breathing or swallowing, shortness of breath, or lip or tongue swelling. There was no history of previous medication or food allergy.

The patient was hemodynamically stable. Her blood pressure was 126/68 mmHg, heart rate 80 beats per minute, respiratory rate 16 breaths per minute, and temperature 98.6 °F (37 °C). The initial exam demonstrated a faint, lightly erythematous, flat, macular rash of the cheeks, ears, and posterior thighs. There was no lip or tongue swelling. Her lungs were clear. She was treated in the ED with diphenhydramine 25 mg intravenously, dexamethasone 6 mg intravenously, and omeprazole 20 mg intravenously, with nearly complete resolution of symptoms, and was discharged from the ED.

**Figure 1:**
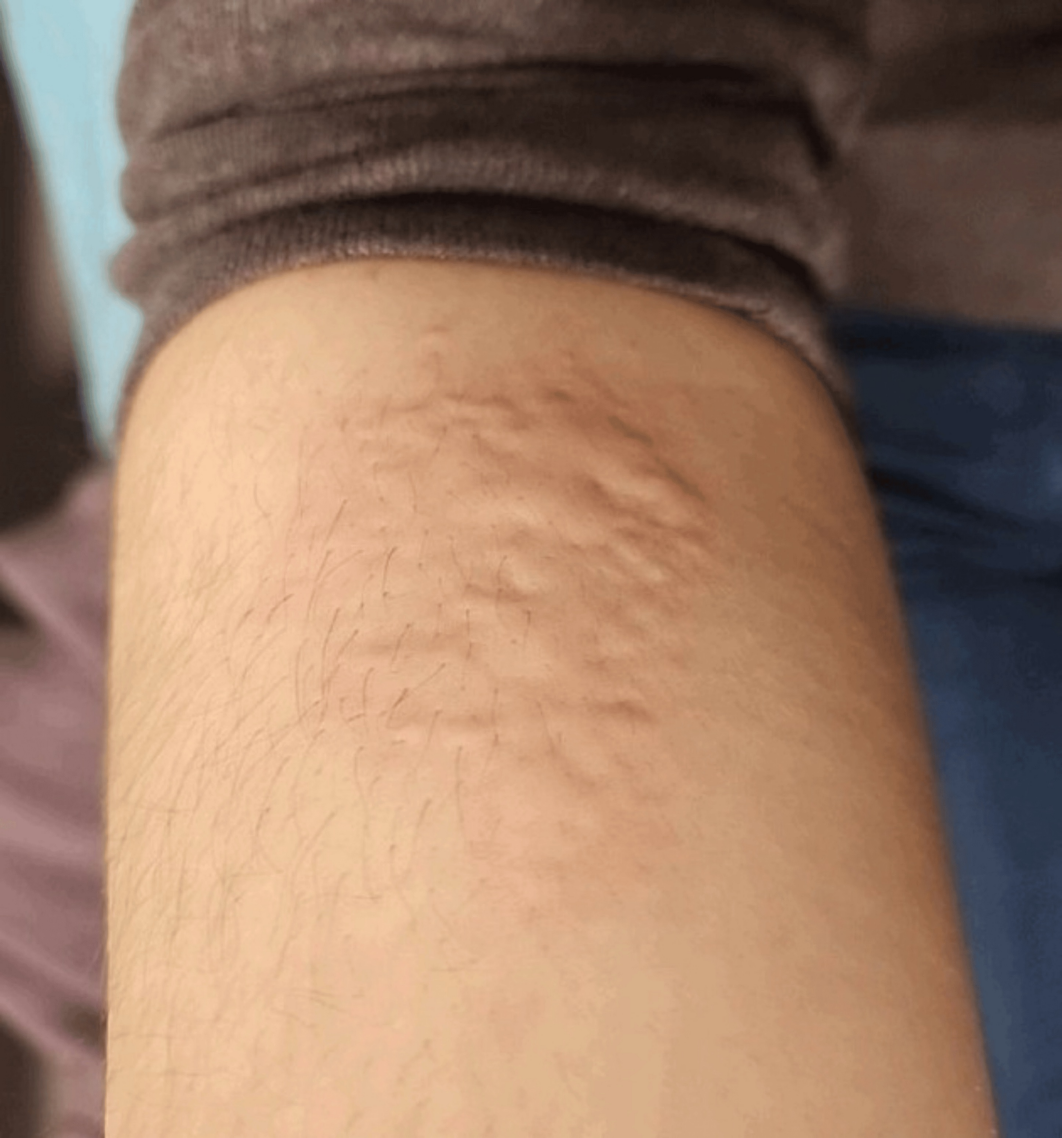
Patient's forearm after cold stimulation with an ice cube

While the patient was standing outside for about 10 minutes awaiting her ride home in the cold weather (around 20 °C on the day of the ED evaluation), her original symptoms returned. She developed an urticarial rash on the neck, posterior thighs, and cheeks. She also developed swelling in her ears and hands, which were both red. She was given an additional dose of diphenhydramine 25 mg intravenously as well as methylprednisolone 125 mg intravenously. She once again demonstrated complete resolution of her symptoms. The provider caring for the patient performed a cold stimulation test in which ice was applied directly to the patient's right forearm and held in place with the examiner's hand. After 10 minutes, the patient developed a hive in the area of the test (Figure 1).

The patient was diagnosed with cold urticaria. Upon further questioning, she explained that she is from an area with a consistently warm climate, and this was the first winter she had spent in an area with cold weather. She also stated that her house, where she is staying, was very cold, which is where the rash initially developed.

The patient was discharged with a follow-up with an allergist/immunologist. She was discharged with instructions for the use of diphenhydramine orally as needed and with prednisone 20 mg twice a day for three days. An EpiPen was prescribed.

## Discussion

The mechanism behind the pathogenesis is not completely known. It appears that cold-induced autoallergens lead to the formation of IgE antibodies against those autoallergens. Mast cells are activated and release histamine and other mediators of inflammation [4,5].

The history and physical exam, including a cold stimulation test, support the diagnosis. For initial localized cases, a history and physical examination are sufficient. Recurrent or severe cases may prompt laboratory evaluation, including cold agglutinins and cryoglobulins, as well as other tests as prompted by the history and physical examination [1].

For mild cases, treatment is essentially symptomatic and involves non-sedating histamines. The sequence of medications beyond antihistamines varies with the patient [6].

Patient education concerning avoiding cold aquatic activities is important because such exposure is more likely to lead to more severe reactions, such as anaphylaxis. ED management of mild cases may include steroid administration. Several sources refer to the consideration of the use of omalizumab in chronic cases. Some chronic cases resolve completely over time [4].

A scoring system has been developed in order to track response to treatment [7]. In reference to prognosis, in pediatric patients, the presence of an elevated eosinophil count was associated with a higher risk of cold-induced anaphylaxis [8]. In adult patients, generalized urticaria and angioedema were associated with a higher risk of severe systemic reactions [9]. Patients at higher risk of serious reactions may require higher doses of second-generation antihistamines [10]. Several possible therapies have been researched in higher-risk and antihistamine-refractory patients, including cyclosporine, montelukast, and omalizumab [11].

## Conclusions

Cold urticaria can be primary (idiopathic) or due to an underlying disease. Most cases are idiopathic. Underlying diseases in secondary cold urticaria include malignancies, infections, and autoimmune disorders. The reaction can be triggered in individual cases by exposure to cold objects or to generalized cold ambient temperatures, as was the case in the patient presented here. The physical response is most commonly pruritic wheals (urticaria). However, more severe symptoms may occur, up to angioedema with hoarseness and wheezing. This patient had mild symptoms, affecting the skin only. Treatment is essentially symptomatic for mild cases, involving non-sedating histamines. Anaphylaxis is treated as indicated. ED management of mild cases may include steroid administration. Several sources refer to considering the use of omalizumab in chronic cases. The emergency physician should perform a full examination, looking for any evidence of severe reactions, such as anaphylaxis.

Patient education concerning avoiding cold aquatic activities is important. Follow-up with an allergist or immunologist should be considered. The literature notes areas for future research, including better ways to identify genetic subtypes and clearer science concerning treatment for various types of cold urticaria.
